# Conceptualising and Understanding Artistic Creativity in the Dementias: Interdisciplinary Approaches to Research and Practise

**DOI:** 10.3389/fpsyg.2018.01842

**Published:** 2018-10-03

**Authors:** Paul M. Camic, Sebastian J. Crutch, Charlie Murphy, Nicholas C. Firth, Emma Harding, Charles R. Harrison, Susannah Howard, Sarah Strohmaier, Janneke Van Leewen, Julian West, Gill Windle, Selina Wray, Hannah Zeilig

**Affiliations:** ^1^Created Out of Mind, Wellcome Collection, London, United Kingdom; ^2^Salomons Centre for Applied Psychology, Canterbury Christ Church University, Canterbury, United Kingdom; ^3^Dementia Research Centre, UCL Institute of Neurology, University College London, London, United Kingdom; ^4^Centre for Medical Image Computing, Department of Computer Science, University College London, London, United Kingdom; ^5^Living Words, Folkestone, United Kingdom; ^6^Royal Academy of Music, London, United Kingdom; ^7^Dementia Services Development Centre, Bangor University, Bangor, United Kingdom; ^8^UCL Institute of Neurology, University College London, London, United Kingdom; ^9^College of Fashion, University of the Arts London, London, United Kingdom

**Keywords:** dementia, creativity, dance, visual art, music, poetry, psychophysiology

## Abstract

Creativity research has a substantial history in psychology and related disciplines; one component of this research tradition has specifically examined artistic creativity. Creativity theories have tended to concentrate, however, on creativity as an individual phenomenon that results in a novel production, and on cognitive aspects of creativity, often limiting its applicability to people with cognitive impairments, including those with a dementia. Despite growing indications that creativity is important for the wellbeing of people living with dementias, it is less well understood how creativity might be conceptualised, measured and recognised in this population, and how this understanding could influence research and practise. This paper begins by exploring prevailing concepts of creativity and assesses their relevance to dementia, followed by a critique of creativity and dementia research related to the arts. Perspectives from researchers, artists, formal and informal caregivers and those with a dementia are addressed. We then introduce several novel psychological and physiological approaches to better understand artistic-related creativity in this population and conclude with a conceptualisation of artistic creativity in the dementias to help guide future research and practise.

## The Dementias and Creativity

The terms “creativity” and “dementias” are not two words that often find themselves linked. When asked about what word comes to mind when thinking about the dementias it is rare if not unheard of for creativity to be identified (Brotherhood et al., 2017; van Leeuwen et al., 2017a). Part of this disconnect is the result of years of creativity research that has focused on eminent creators in science and industry, university undergraduate psychology students as part of a course requirement, artists of various sorts, and gifted “geniuses” with very little research exploring creativity and people with mental or physical health problems, the exception being the apparently “mad” artist (e.g., Csikszentmihalyi, 1997a; Chad et al., 2007; King Humphry, 2010; Bellas et al., 2018). The development of the “mini and little c” creativity models (Kaufman and Beghetto, 2009), among other recent advances which we will address allows for a more extensive exploration of creativity across different physical and mental health conditions. This paper examines the concept of artistic creativity and the dementias with an aim to encourage researchers, practitioners and policy makers to generate more research, enact arts and health policies and develop arts and dementia care programmes to help shape dementia care internationally.

### Brief Overview of the Dementias

Recognition of the dementias *(pl.)* and their earliest impacts has been slowed by traditional definitions of dementia which emphasise impairment of memory and criteria which require cognitive impairment sufficient to compromise social and occupational functioning (American Psychiatric Association, 2000). Many diseases can result in a progressive dementia syndrome. The most common causes both in the elderly and in younger people are Alzheimer’s disease (AD), vascular disease, frontotemporal lobe degenerations (FTLD), and dementia with Lewy bodies (DLB). A number of dementias are associated with particular symptom profiles (e.g., DLB: hallucinations, cognitive fluctuations and Parkinsonian gait; semantic dementia: impaired language comprehension and semantic memory). However, heterogeneity in the dementias is increasingly acknowledged, with contemporary Alzheimer’s disease criteria describing not only the classical amnestic presentation, but also atypical presentations affecting visual perception, language or behaviour/executive functions (McKhann et al., 2011; Dubois et al., 2014). Atypical presentations and rarer dementias highlight the range of cognitive skills which may become vulnerable in anyone with a dementia as the condition progresses. Equally this heterogeneity serves to underline the relative preservation of certain skills and abilities well into a disease course when other aptitudes may be perceived to be profoundly compromised. It is against this complex, evolving cognitive background that different forms of individual and collective creativity in people with dementia must be considered.

### Prevailing Concepts of Creativity and the Dementias

The idea of creativity is surprisingly recent. As Pope (2005) argues in his historical and critical guide to the concept the first recorded usage of creativity in English occurs only in 1875. Thus, the emergence of the concept coincided with the late Romantic period and was closely associated with the arts (Williams, 1988) and with the notion of the “artistic genius.” Even recent conceptualisations from both psychological and neurological perspectives tend to link creative processes to specific, original and tangible acts of production that are associated with individual motivations (e.g., Csikszentmihalyi, 1997a; Palmiero et al., 2012).

These are of relevance in that the myth of the “creative individual”, the “genius,” is a powerful motif shaping social understandings of creative activities (Runco, 1987). This hegemonic narrative not only informs shared ideas about age and creativity (McMullan and Smiles, 2016) but of central relevance for our discussion here, also influences the ways in which notions of creativity relate (or more pertinently do not relate) to people living with a dementia. Focusing on the characteristics and capacities of an individual defined as particularly creative, the narrative understands creativity as something psychologically inherent to a creative individual (Osborne, 2003). Recognising creativity and the production of creative acts as collective as well as individual (Becker, 2004) and also associated as much with process as product (Plucker and Beghetto, 2004), we explore the opportunities and constraints that are experienced by people living with a dementia in a variety of contexts and the ways in which these may extend our understandings of artistic creativity. The ways in which social practise (i.e., how individual and contexts codetermine each other) are situated or how central cognition seems to be in our understanding of creativity, are not fixed (Barb and Plucker, 2002, p. 169) but part of an ongoing debate about how to define creativity. Locating creativity primarily as a cognitive domain limits, however, the applicability of creativity as a construct in dementia research and care. As cognitive capacities decline and become less and less accessible it is important that researchers and clinicians do not assume that the potential for creative activity is eliminated.

The absence of a precise definition of a concept such as creativity can be problematic for research but arguably, it may also be that a universal definition of creativity and specifically, creativity and the arts, limits its applicability across people and environments and a more situated perspective is necessary (Clarke et al., 2018). For example, there are aspects of the definition offered by Plucker et al. (2004) that fit well across dementias (process, environment, and social context) but one aspect, aptitude, does not; the latter not necessarily being salient to everyday artistic creativity for this population. Whilst an in-depth review of the multiple prevailing definitions of creativity is beyond the scope of this article, four appear highly relevant to conceptualising the arts, creativity and dementias.

In an attempt to incorporate cross-cultural variations in Western and Eastern perspectives the four-criterion construct of creativity (Kharkhurin’s, 2014) uses the attributes of novelty, utility, aesthetics and authenticity to develop a matrix to compare creative products from “different areas of human endeavour across the arts, sciences and business” (p. 349). Two of the components resonate well with dementias. Utility refers to creative work perceived as such by the producer of the work and the recipient, producing a landmark in social or cultural environment and addressing moral issues. Secondly, authenticity, taken from Confucian aesthetics, is particularly noteworthy and reflects a process of bringing new responses into existing ideas to reflect an individual’s own essence at a moment in time (Tu, 1985). These components expand the concept of creativity to include the role of a socio-cultural context, individual perceptions and responses from others that build on a more inclusive concept of creativity beyond cognitive factors.

Drawing on Rowlands (2010) ideas of an embodied, embedded and extended mind, Glăveanu (2013) sought to situate and contextualise creativity and developed the five A’s framework which, he argues, represents “a fundamental change of epistemological position. In light of sociocultural sources, the actor (creator) exists only in relation to an audience, action cannot take place outside of interactions with a social and material world (affordances), and artefacts embody the cultural traditions of different communities” (p. 71). This framework is relevant to our discussion in that it outlines the inherently interrelated nature of the various aspects of creative endeavour. Above all, his framework places the creator (in our work the person with a dementia) in a broad context of material, social and cultural phenomena and relations. Glãveanu’s framework represents a more fully systemic and situated theoretical model for understanding contextually how and when artistic creativity might take place across the spectrum of the dementias. For example, a person putting several words together poetically in an advanced stage of Alzheimer’s disease as an expressive response to listening to music in the context of receiving residential care, could easily be minimised as a chance event. Yet, given that this person may not have spoken for months her poem might provide insights into her experience of living with dementia and can be understood as a creative response *at this point in her life*.

Glăveanu (2013) and Kharkhurin’s (2014) contributions also blend well with the concepts of little and mini-c creativity, introduced by Beghetto and Kaufman (2007) and Richards (2007), respectively, which is the third perspective we draw upon. Little-c creativity, also referred to as “everyday creativity,” results in creating something new that has originality and meaningfulness (Richards, 2007) and mini-c creativity is “the novel and personally meaningful interpretation of experiences, actions, and events” (Beghetto and Kaufman, 2007, p. 73). Although appearing quite similar, mini-c creativity is an internal process that consists of ideas and connexions that may not always be visible to anyone except the creator and can be challenging to measure, understand and value in the dementias.

A fourth perspective that contributes to our understanding of creativity and dementias is the heuristic approach proposed by Batey’s (2012), which is oriented toward developing a framework for measuring creativity across three axes: the level to be measured (e.g., individual, group, community), the facet of creativity to be assessed (process, press, product, and trait) and the measurement approach (e.g., objective, self-rating). The inherent flexibility of this framework offers the possibility of developing longitudinal research; it fits well across different types of dementia, addresses challenges in measuring and understanding creativity as impairment increases over time and takes into consideration changes in the home, community, hospital and residential environments, (e.g., settings). Batey’s approach also provides a useful measurement strategy that can be used across the three frameworks cited above (Beghetto and Kaufman, 2007; Richards, 2007; Glăveanu, 2013; Kharkhurin’s, 2014).

### A Snapshot of Dementia and Creativity Research

Over the past 10 years, there has been an increasing interest in research on dementia, the arts and creativity across different disciplines (Palmiero et al., 2012). Creative expression in artistic activities such as painting or making music, for example, has been found to be an important way for people with a dementia to express and access emotions even when cognitive abilities are diminishing (McLean, 2011; Zeilig et al., 2014). Rather than as a form of treatment for cognitive decline, creative activities involving the arts are often used in the context of therapy as part of the treatment of behavioural and emotional problems in dementias (Cowl and Gaugler, 2014). Previous research argued that art therapy was a potentially beneficial non-pharmacological intervention for dementia to improve quality of life (Mimica and Kalini, 2011). However, optimal conditions in the design of art interventions for the dementias to foster creativity need to be identified (Chancellor et al., 2014). This was reflected in a recent review of studies on art therapies and dementia revealing incoherent methodologies and tools used to assess creativity where a majority of studies focused on and judged the final product (e.g., Joy and Furman, 2014), for instance a completed picture or other artwork, rather than the process of engaging with the creation of art (Crutch et al., 2001; Beard, 2011).

Different forms of arts-based creative expression have been adopted for dementia populations (e.g., visual art making, playing music and singing, storey-telling, poetry). Ullán et al. (2012), examining an art making educational programme for people with mild to moderate dementia, discovered that participants showed surprised satisfaction at being capable of making art and of having created something with their hands, which appeared to reinforce a more positive self-image. Additionally, in a blocked randomisation design with individuals with moderate to severe dementia engaging in singing, listening to, and creating music, a reduction of agitated behaviours was observed during the intervention as well as at 1-month follow-up (Lin et al., 2011). Finally, (Fritsch et al., 2009) through randomised matched pairs incorporated storytelling as a creative intervention with nursing home residents with dementia and their carers, and discovered, compared to the control group, those using the creative intervention showed significant increases in pleasure, engagement and alertness, interacted more with nursing home staff, and socialised more. In a follow-up study with the same intervention, significantly improved communication skills both with carers and peers were also observed in people with a dementia who had participated in a creative expression intervention through storytelling (Phillips et al., 2010).

In a review of studies and case reports on creativity in dementia, Palmiero et al. (2012) discovered that although people with dementia were generally found to be able to express artistic creativity, divergent thinking was considered to be affected in both artistic and non-artistic people with a dementia in the sense that those with a dementia were found to be less inventive in creating novel art products. For instance, previous research observed alterations in visual art productions in individuals with different forms of dementia and although drawings by individuals with Alzheimer’s disease were closest to drawings of healthy controls, individuals were found to use more muted colours and included fewer details (Rankin et al., 2007). However, Ullán et al. (2012) argue that more simplistic forms of artistic expression do not necessarily mean less creativity.

Furthermore, creativity and creative expression have been found to look different depending on the type of dementia and its corresponding area of the brain as well as the context of the creative activity. Based on a review by Gretton and ffytche (2014), it appears possible that a unique artistic signature exists for each type of dementia diagnosis with different expressions of creativity in visual art depending on the area of damage in the brain. Research looking at creativity and dementia with Lewy bodies (DLB), which examined drawings of a visual artist before and after the onset of the dementia, discovered a gradual decline in all artistic qualities except for novelty as the disease progressed (Drago et al., 2006). Art produced by individuals with semantic dementia has previously been described as being “bizarre” and “distorted” and failing tests of divergent thinking (Rankin et al., 2007). Lower ability levels for creative expression have also been identified in individuals with a diagnosis of frontotemporal dementia (Joy and Furman, 2014) due to degeneration of the frontal and temporal regions of the brain. According to de Souza et al. (2010; p. 3733) any form of artistic expression is thought to be due to “involuntary behaviours” rather than as an expression of purposeful creativity. However, the question arises, even though artistic expression changes after onset of dementia, does this imply a reduction of creativity *or* a different form of creativity? Likewise, what type of creativity is being considered? For the purposes of this paper, we are interested in understanding everyday artistic creativity (Richards, 2007), most decidedly being of the little-c or mini-c variety where the focus is on the non-expert (Kaufman and Beghetto, 2009).

### Co-creativity – Mapping the Concept

Like artistic creativity in people with dementias “co-creativity” is a nascent concept that has yet to be fully theorised. Nonetheless the term is steadily gaining in popularity, indeed the closely allied phrase “co-creation” can be found in various contemporary media (Zeilig et al., 2018). However, there is currently no agreed definition of co-creativity and therefore the concept itself remains somewhat indistinct. The emphasis in business and design contexts is upon the transfer of value from an end (or predefined) product to a shared process in which all those involved play an integral role in bringing something that is mutually valued into existence (Branco et al., 2017).

Artistic co-creativity as theorised and practised with people with a dementia shares some similarity to the understandings offered by design and business, in particular the possibility that distinctions can be erased between the artist-producer and participant-artist (Zeilig et al., 2018). Equally, the emphasis on the equal contribution of all involved is pertinent. However, it fundamentally differs conceptually in that the objective is not to co-design a product or work toward a single composition or performance. The work of Matarasso (2017) has been informative here. He similarly discusses co-creation in the context of arts-based projects and how artists do not instruct but rather disperse the authority associated with their skills, thereby privileging the creative process over an end product. However, this is not to imply that lone creativity does not also involve intense and embodied engagement with the processes of creating. As cogently outlined by Banfield and Burgess (2013) in their reconceptualisation of Csikszentmihalyi’s (1997b) “flow” experience within artistic practise, process is key for individual artists too. These authors suggest that flow, an integral part of the creative process, is particularly important for visual artists who work in two dimensions (Banfield and Burgess, 2013, p.74). The distinction in terms of co-creativity is that creative process and allied experiences of flow are more likely to be shared between two people or by multiple people at group events.

Thus, although there is not currently a single agreed definition for co-creativity it is characterised by a number of key features including centrally, a focus on shared process, the absence of a single author (hence unity and shared ownership), inclusivity, reciprocity and relationality. Co-creativity relies on dialogic and empathic approaches (Sennett, 2012) where through the process of exchange, understandings are expanded, although not necessarily resolved. This is in contrast to dialectic encounters which tend to lead to closure (Sennett, 2012, p. 24). Above all, it contrasts with notions of the lone creative genius that have tended to dominate views of creativity.

The role and value of the creative arts for people living with a dementia has been widely appreciated (Young et al., 2016; Camic et al., 2017; Windle et al., 2017), yet it has not explicitly focused on the ability of people with a dementia to interact and engage as co-creators. This may also reflect different disciplinary aims and theoretical perspectives, and the location of the majority of theories of creativity within a cognitive framework (Plucker and Beghetto, 2004) but may also be linked with dominant perceptions that people with a dementia are less capable of creative interactions (Basting and Killick, 2003; Ullán et al., 2012). There is thus a nascent but steadily growing recognition that people living with a dementia may be able to engage co-creatively with the arts (Kontos et al., 2017, p. 188).

“…*individuals with dementia can make recognisably creative contributions despite the absence of sensical language*.”

Co-creativity using the arts extends an invitation to participate in an aesthetic process and allows unique opportunities for communication and expression. The possibility that co-creativity can challenge the dominant biomedical perspective that associates the dementias with irretrievable loss and decline by creating opportunities for creative agency is a foundational premise of the projects presented below. As a process and as a tool or strategy for self-actualisation, in which micro-acts of artistic creativity gain significant importance within a group setting, co-creative activity may therefore be positively associated with the maintenance and promotion of various aspects of health and wellbeing (Price and Tinker, 2014) as well as providing important opportunities for playfulness and fun.

## How Do People With A Dementia Perceive Creativity?

A search of the literature revealed no studies that examined how people with a dementia perceive and appreciate *their own* artistic creativity. We have found this omission to be problematic in that creativity has become defined by others (e.g., researchers, clinicians, the general public) without taking into consideration the perspectives and experiences of those living with a dementia. One recent systematic review (Nyman and Szymczynska, 2016, p.104) identified the pursuit of new leisure activities (including the arts) as a way for people with dementia “to avoid becoming stagnant…and to create a new path…(whilst) leaving a legacy for younger generations,” yet absent was how those with a dementia value or understand their own creativity. Although changing, the perspectives of people with a dementia have historically not been taken into consideration when planning services or undertaking research (Wilkinson, 2001). Any conceptualisation of creativity and dementia, we argue, needs to take into consideration the perspectives of those with a dementia along with caregivers, both formal and informal. As part of the development of our understanding of creativity and the dementias we felt it essential to seek the perspectives of people with a dementia and caregivers about this topic. In preparation for this article the authors sought to broaden their understanding of artistic creativity and the dementias beyond the research literature by having a series of conversations with people with a dementia and caregivers. Not designed as a research project that sought to generate new data, the following questions helped to form our conversation: What does creativity mean to you in your day to day life? How do you personally understand artistic creativity? How does creativity impact dementia and how does dementia impact creativity? Is creativity always something positive, and if not, when is it not positive? **Supplementary Table S1** provides a sample of responses, which along with previous and ongoing research, have contributed to our conceptualisation of how artistic creativity is experienced by those with a dementia and caregivers.

## Creativity In Context

Over a 2-year period (2016–2018) the authors, an interdisciplinary group of researchers, artists and media professionals, have been involved in a series of art experiments at Created Out of Mind^1^ a Wellcome Trust funded project examining the potential of different art forms and cultural activities to help better understand the experience of the dementias and likewise, to appreciate how the dementias might influence our understanding of artistic creativity. This section reports on several of those ongoing and novel initiatives and presents new methodologies that have not yet been used in creativity and dementia research. These diverse projects occurred across different dementias and levels of impairment in community and residential care settings as well as in more traditional laboratory environments and in public forums. All projects have been ethically reviewed and approved by faculty ethics panels at either University College London or Canterbury Christ Church University. Some of these projects have been presented at conferences, others will be written up for journal articles whilst others are early days research that will be further developed.

### Creative Opportunities in Dementia Care Environments

About one-third of people with dementia live in residential care and approximately two thirds of people who live in care homes are thought to have dementia (Department of Health, 2013). Care homes face many conflicting pressures involved in delivering day-to-day care, often described as task focussed, and despite best intentions, there is often limited scope for staff and residents to engage in meaningful activities together. Although problems in measuring creativity in this environment are pronounced, nevertheless, there is a growing recognition of the capacity of care homes for establishing artistic/creative residency programmes. In many instances this is motivated by a wish to improve the quality of life of those living with dementia (e.g., Cutler et al., 2011) and there is increasing evidence supporting the role of the arts across a range of positive outcomes (e.g., Windle et al., 2016).

### Co-creativity and Advanced Dementia

Helping to provide a stimulating and creative caring residential environment for those with advanced dementias has often been overlooked or simply not considered as part of national dementia care policies (All Party Parliamentary Group on Arts, Health and Wellbeing [APPG], 2017). The practise of *Music for Life* founded in 1993 by Linda Rose has, however, placed a particular emphasis on working with people with advanced dementias. The intention to create community and shared experience through the use of musical improvisation has many parallels with a co-creative approach and is framed by both mini- and little- c creativity (Kaufman and Beghetto, 2009). By improvising pieces of music together (the genesis of creative expression as described through mini-c creativity) professional musicians, people with advanced dementias and professional care staff are engaged in musically responding to each other through what we have labelled as taking creative risks (e.g., picking up an instrument and playing for the first time; conducting the group for a brief period of time; responding musically to a musician or other group member). As dementias progress, many but not all (e.g., those with frontotemporal lobe type dementia) may lose confidence, interest and optimism in their abilities. Attending an arts group where everyday creativity (little-c creativity) and interaction with other members and facilitators is encouraged, may need to be gradually introduced in order to reduce anxiety and encourage participation and creative risk taking. In doing so members have the opportunity to relate to one another in ways that they might not do so usually and beyond the usual restrictions of their perceived roles. By shifting the emphasis onto relationship and communication processes rather than achieving a specified outcome, an ability and desire to engage in mutual exchange is revealed. In the project, *Music for Life 360*, several novel technologies were used to capture psychophysiological information, through wearable data collection devices (see section “Psychophysiological Responses to Creativity for People Living with Dementia”), and group interaction processes recorded through 360-degree video cameras (360fly, Canonsburg, PA, United States). The use of a 360-degree camera allows simultaneous interactions to be captured and later more fully understood through slowed-down (0.25 s per frame) video analysis using a software programme. This has enabled greater clarity in ascertaining the extent to which people living with advanced dementias are responding to co-creative interactions, whereas observational methods are more influenced by vocal and motor responses and possible biases of observers (Zeilig and West, 2017). The question of whether or how moments of shared creative experience affect us, regardless of our stage of life and cognitive ability, is addressed. Indeed, the idea that highly trained professional musicians might be stimulated and influenced by their creative interactions with people with advanced dementias could be a meaningful illustration of the concepts of creative and relational agency where the creators are interdependently engaged with a social and material world within a cultural context of artefact production (Glăveanu, 2013). The artefact production in this context (singing) is *both* process and product.

### Residential Caregiver Involvement in Creative Activity

Equally important, professional caregivers’ experiences of creativity in practise is a powerful tool toward enhancing care quality. These can enhance client-carer interactions, validating the personhood in residents with dementia (Broome et al., 2017). For example, Basting et al. (2016) describe how they enacted a depiction of *The Odyssey* in the day-to-day running of care facility. This engaged residents, staff and family members in a uniquely creative way to improve quality of life and showed how the arts can transform environments.

Working to reach socially isolated residents within the care environment (e.g., bed bound, those displaying distressing behaviours), one such programme, *Living Words*, developed a 7-stage residency process. Residencies to date have taken place in 24 residential settings, with 820 participants and include using the “listen out loud” method (Gardner, 1983) to co-create an individual book of poetry with each participant focusing on their emotional experiences rather than cognitive abilities, which may vary greatly across participants.

Influenced by Kaufman and Beghetto (2009) mini-c (“genesis of creative expression,” p. 2), Richards (2007) everyday creativity (little-c creativity) model and Batey’s (2012) heuristic framework, creativity was explored through relationship building and the process of constructing poems together. As an example, Sherman was known to shout and interrupt people, banging his fist on a table. Artists were told that he was “incoherent” and had “challenging behaviours”. Through working with a *Living Words* (2014) artist who wrote down and then read his words to him, he began to express his feelings: “I am scared… I don’t know where I am.” The validation of his emotions, words and even the fist banging led to him verbalise more (mini-c creativity), while his banging and shouting lessened. This creative relationship enabled staff to better understand Sherman the person, rather than just his dementia. This supports previous findings that through creativity in dementia, “feelings of peace may be generated” Zeilig et al. (2014, p. 26).

Another resident, Sally, spoke very quietly and in metaphor. This made it hard for staff to hear and understand her. On seeing the *Living Words* book she co-created and hearing her words read to her, staff reported being able “to see” the meaning in her words. For example, staff realised when Sally spoke of machines she was talking about brains; when she informed them that “the world is talking” she was referring to the care home. Sally’s voice became louder and she expressed joy in sharing her book, “One becomes a little more alive … Not just hanging there.”

### Profiles in Paint and Single Yellow Lines

Taking a brush to canvas is an artistic activity available to most people with a dementia, regardless of previous visual arts experience. Guided by Glãveanu’s 5-A’s framework, one method we have begun using to capture artistic creativity through painting, in the context of different dementias, has been to invite people with an interest in art-making to arrange a group of 12 objects and independently produce a still life painting of their arrangement. The first exploratory study, *Profiles in Paint*, involved four people with different diagnoses of dementia, behavioural variant frontotemporal dementia (bvFTD, primary progressive aphasia (PPA), posterior cortical atrophy (PCA), typical Alzheimer’s disease (tAD) and a control group of four people without a diagnosis (Harrison et al., 2017). All artists received the same materials and instructions and the procedural framework allowed comparisons to be made between the works. For example, the artist with bvFTD approached the exercise in a way that accentuated their individual artistic interests whilst the artist with PPA created a structure to communicate relationships between the objects. The artist with PCA and the artist with tAD both found some of the objects perceptually challenging but this also allowed for a greater focus on the sensual qualities of the medium. Giving people with a dementia a choice over object arrangement also allowed a cooperative interaction to occur with the researcher that facilitated further understanding of perceptual, emotional and motivational aspects of creativity.

Since 2016, the *Single Yellow Lines* project has been examining the creative potential of painting a line. Initially 55 people who attended Rare Dementia Support Groups (PCA/PPA/FTD) were invited to paint a straight line on one canvas and a line of their choice on a second canvas. A further 99 people without a dementia at public events have painted their own straight and expressive lines. The straight lines are initially being examined in laboratory and cultural venue environments as a potential measure for the spatial disruptions people with PCA experience. However, it is interesting that due to the decentralisation of perceptual experience associated with PCA, the expressive lines made by people with this diagnosis have also appeared the most expressive to many observers (e.g., neuropsychologists, artists, general public). For people whose verbal language skills are compromised the expressive line may also offer opportunities to communicate in another form, using images, words or metaphors.

We are continuing to investigate if the paintings made in these projects may be indicative of common symptomatic features of different dementias. Through public engagement events we have also observed how paintings have been powerful tools for communicating different experiences of the dementias to diverse audiences, ranging from neuroscientists to the general public. The projects aim to broaden the debate on the concept and manifestation of creativity in the dementias and seek to challenge the assertion that definitive interpretations about artistic creativity can be made in relation to diagnostic criteria. As with some definitions of creativity discussed earlier, it is perhaps in the *process* of creating that is felt most intensely (mini-c creativity) and because of this, the pleasures that are manifest in painting are not necessarily compromised in the context of a dementia.

### The Neuronal Disco: Dancing Connexions Between Art and Science

Creativity research also has a role to play in conveying scientific complexities in dementia research to a wider audience outside of academia. One area of this research looks at how artistic responses to various aspects of brain abnormalities can offer audiences new insights into the mechanisms supporting the growth and degeneration of brain cells. For example, in order to investigate why abnormalities in the protein tau can lead to neuronal death in familiar Alzheimer’s disease (fAD) and frontotemporal dementia (FTD), fibroblasts (skin cells) generated from participants carrying genetic mutations linked to disease are reprogrammed into induced pluripotent stem cells (iPSC). These iPSC can subsequently be differentiated into any cell type of interest, including neurons, which can be grown in both 2D and 3D culture formats (Arber et al., 2017). Comparisons between the neurons grown from participants with and without dementia can then be used to understand the earliest changes in disease cultures.

Grounded in Kaufman and Beghetto (2009) “Four C Model,” a new component of this research also investigates how researchers and artists might effectively convey scientific information (Big C-creativity) through creative activities with people living with FTD and Familial Alzheimer’s (fAD) as well as reflecting on the profound personal, ethical and metaphysical implications that these technologies present. As part of an initial pilot study a visual and performance artist began to consider how she could represent and embody (Pro-C creativity) what was growing in the laboratory in a form which would dynamically convey the earliest stages of cellular change and encourage public dialogue and discussion about the dementias. Researching ways to animate each change and structure of the cell development through choreographed formations of growth and degeneration, identified music, movements and groupings which could express different morphologies of dementias through a kind of cellular hybrid of country and disco dancing. The resulting *Neuronal Disco* (little-c), was subsequently trialled as a form of public engagement dance initiative to encourage people of different ages to discuss dementia (Murphy and Wray, 2016).

Devised initially as a creative exercise to better understand these cellular processes, the *Neuronal Disco* evolved into a playful participatory event intended to engage public audiences in the science and aims of this research. Artist and scientist team leaders guide participants through each stage of the research in a series of choreographed groupings which mirror cellular mechanisms and transformations at different scales, performing axonal transport using illuminated balloons as vesicles and coloured streamers to create neuronal networks and tangles (mini-c) (Murphy and Wray, 2016). Appropriating rituals and accessories from rave and party contexts, participants were invited to wear small lights placed on all five fingers in colours matching the stains used to identify particular proteins, while their sound and light bracelets lit up in response to themed music (Wray and Murphy, 2017).

The *Neuronal Disco* invites a broad audience to consider the impacts of dementia on a molecular level through playful physical enactments. Abstract laboratory-based processes (mostly off limits to the public) are transformed into accessible group interactions which are informed by the laboratory team’s perspectives (who perform this work on a day to day basis) and the artist’s perspective (who has observed her own cells being transformed).

Performing each stage of the research together as a group helps us to creatively interpret and conceptualise the molecular dimensions of dementia research, offers insights into the science behind this research and opens up a new perspective on how we think about and visualise life altering diagnoses. Through the use of public engagement in dancing (mini-c creativity) where no previous dance experience is expected, the general public participates in an enjoyable creative activity as they learn about some of the laboratory science in dementia research. These types of activities also have the potential of shaping public attitudes toward the dementias, lessening stigma and supporting dementia friendly communities (All Party Parliamentary Group on Arts, Health and Wellbeing [APPG], 2017).

## Psychophysiological Responses to Creativity for People Living With Dementia

Understanding creative experiences through psychophysiological measures has the potential to allow researchers to more fully comprehend physiological responses across periods of time, different dementia diagnoses and impairment severity. These measures are not dependent on cognitive ability and can be used longitudinally across the progression of dementia to assess reactions and responses to different art forms (e.g., playing music, poetry, singing, and painting) (Harding et al., 2017) during mini and little-c creative activities in individual and group settings. Psychophysiological measures have been shown to correlate with involvement during creative practise in a wide range of arts activities (e.g., De Manzano et al., 2010; Tschacher et al., 2012; Tröndle et al., 2014). Such measures offer an objective measure of participants’ involvement or engagement in creative practises complementary to more subjective self-report measures such as visual rating scales and interviews, during earlier and middle stages of dementia, and with video recording and other observational tools during later stages when impairment is severe. Recent advances in wearable technology have decreased costs and increased accuracy of unobtrusive devices so that they are now similarly accurate in emotion recognition tasks (Ragot et al., 2017). To better understand psychological and physiological responses to creative arts activities by those with a dementia, wearable technology has been used to continuously measure psychophysiological changes *during and across activities* (Bourne et al., 2017). Empatica E4 wristbands (Empatica, Cambridge, MA, United States), watch-sized devices, were employed to measure the following (Brotherhood et al., 2017):

- *3-axis accelerometer*: Provides information about levels of physical activity.
- *Electrodermal activity (EDA, an indication of arousal)*: A measure of emotional and sympathetic response useful for detection of levels of emotional and physiological arousal.
- *Blood volume pulse (BVP, used to derive heart rate)*: Used as a measure of heart rate, which may indicate excitement, stress and/or increased physical activity.
- *Peripheral skin temperature (an indication of stress):* Similarly, to arousal, a measure of stress for determining level of stress in a wide range of activities.

Due to their high sampling rate wristbands such as the E4 collect vast amounts of continuous data capturing psychophysiological responses during creative activities, which can be collected unobtrusively across community and residential care settings. Because participants appear not to be aware they are wearing the devices this potentially makes these measurements more representative of a creative experience than an experimental condition. The unobtrusive nature of the devices also permits the collection of meaningful levels of baseline data which aid interpretation and analysis.

The interpretation of physiological data is not straightforward. For example, as well as participation, increased activity levels could signal agitation (e.g., fidgeting, attempts to leave the room), and emotional arousal could be positive or negative, and even when negative, this could be an engaged and meaningful response to a challenging artwork, and possibly indicate an embodied form of “flow state” (wide Banfield and Burgess, 2013) or a feeling of disgust accompanied with a desire to withdraw from the activity at hand. Difficulties with interpretation arguably make isolated use of such measures problematic (Thomas et al., 2018). Furthermore, there is far less experimental control and far greater complexity in creative arts activities than in carefully controlled psychology experiments. Ideally such data should be interpreted alongside supplementary observational field notes or video data to re-contextualise moments of physiological activation. The issues of interpretation also raise important questions about hypothesis development of creative involvement and whether such activities are studied and measured with the intention of improving wellbeing, quality of life, levels of emotional engagement or communication between a person with dementia and their family member. Batey’s (2012) creativity framework is useful here to help situate the level, facet and measurement approach. As an objective measure continuous physiological measurement lends itself to examining process over a specified time period in individuals, dyads and groups. It also can be combined with other objective measures and subjective ratings to produce a more comprehensive assessment of creativity.

Issues surrounding interpretation also have a bearing on the analytic approach taken with such data. In the early literature on EDA (previously termed galvanic skin response), heart rate and other measures such as electromyography, the prevailing approach was to hypothesise response increases as markers of anxiety, stress, threat-detection and other tension, (e.g., Darrow, 1936; Dittes, 1957; Fowles, 1980). This has contributed to implicit assumptions that higher psychophysiological markers equate with someone being more stressed, anxious or uncomfortable. Secondly, engagement with the arts or other creative processes is much less clearly delineated as being wholly negative or positive, stressful or pleasant, and it seems that the level and quality of engagement itself would be the most appropriate proxy for any measurement of the quality of the experience; whether that be feelings of great tension while grappling with a new medium or composition choices, increased heart rate when joining an improvised dance or playing a piece to the point of crescendo.

In agreement with the majority of the literature in this field (Thomas et al., 2018), we have found that psychophysiological measures are useful in the context of understanding process responses whilst participating in creative activities (Bourne et al., 2017). In particular, using wearable devices to complement mixed-methods approaches to creative involvement and activity we are able to provide quantitative data to test various hypotheses, some across discrete moments in time.

## Visual Thinking Strategies

Perhaps one of the most valuable aspects of art, in any form, is that it creates an ambiguous space of being able to create in which there are no right or wrong answers. Yet the feeling of getting it wrong is unfortunately an experience many people living with a dementia can often relate to (Batsch and Mittelman, 2012). There is therefore a need for clinical assessments of dementia that minimise creating a sense of failure, taking into account a person’s rich life experiences and looking at their current difficulties as well as their functional capabilities. One way to approach this problem is to investigate the potential of the arts-based facilitated learning method Visual Thinking Strategies (VTS) to help people living with a dementia create meaning through viewing visual art, whilst also promoting social wellbeing and potentially serving as a valuable diagnostic tool for clinicians (van Leeuwen et al., 2017b). VTS lends itself to Kharkhurin’s (2014) four criterion construct of creativity involving attributes of novelty, utility, aesthetics, and authenticity (meaning). It also draws on Batey’s (2012) heuristic framework that provides flexibility in designing research with different measurement approaches, studying individuals within group settings (level), while focusing on the facets of process, trait or press.

Visual Thinking Strategies is constructed as a moderated group discussion which allows people to create meaning based on their personal observations of visual art. The moderator uses clearly described techniques to carefully structure the discussion: (1) asking participants to identify visible references for their thoughts and pointing these out, (2) neutrally paraphrasing each comment, and (3) connecting the comment to the ongoing discussion. In education, neuro-rehabilitation and museum settings, VTS has been shown to improve written and spoken language skills as well as social, observation and critical reflection skills (Housen, 2002; Naghshineh et al., 2008; Miller et al., 2013; Hailey et al., 2015).

We are exploring if VTS can enable people living with a dementia to express their personal experiences and feel socially connected without relying on memory or previous knowledge. The ideal context for a VTS conversation is a small group setting with the art object present in its original form and viewed under optimal lighting and spatial conditions. However, in order to operationalise the complex interaction between social context, visual thought processes and moderating techniques at play in VTS a computer-based eye tracking paradigm (Isaacowitz et al., 2006) has been designed to monitor these interactions. People are shown visual artworks and complex images on a computer monitor and the eye-tracker records what their eyes are looking at and in which order eye movements occur. In separate experiments people are shown each artwork for various amounts of time. In one experiment they are being played audio recordings of other people reflecting on the artworks while they are looking, in another they are being asked to personally reflect on the artworks with the 3 VTS questions. The focus of this novel method is on *how* people create personal meaning in relation to what they see, hear and communicate. This approach allows people to express themselves freely, lessening the concern they are getting it wrong, often a commonly voiced concern of people with a dementia.

The ultimate aim of this methodology is to harness its findings into guidelines for cultural VTS programmes tailored to people living with a dementia as well as developing a validated diagnostic assessment tool for clinicians, which lessens the distress and discomfort often experienced in current neuropsychological assessment.

## Conceptualising Creativity In The Dementias

Creativity research in psychology has a long history of being constructed through a cognitive lens; we argue that this is problematic for those with a dementia and others with neurocognitive disorders because it potentially devalues their capacity to be creative. As cognitive capabilities decrease it is essential to examine situational, social and environmental components—in addition to *or instead of* cognitive components—to better understand the value of mini and little c models of creativity (Kaufman and Beghetto, 2009) and they might pertain to people with a dementia (Plucker and Beghetto, 2004; Palmiero et al., 2012; Young et al., 2016). Even as artistic expression may change over the course of the dementias (Crutch et al., 2001), and as cognitive abilities decline, there remain possibilities for artistic creativity to develop. Moreover, as Ullán et al. (2012) noted, simpler forms of artistic expression should not be equated with a lower level of creativity. Whilst there may or may not be reductions in creative activity in a specific art form (e.g., oil painting, glass blowing, ballroom dancing) during any phase of the dementias, this does not imply that alternative forms of creative activity cannot be developed.

The cognitive dominance in creativity research has been reinforced by theoretical assumptions that are not always applicable to this population. Quantitative approaches to creativity often involve measuring levels of memory, motivation, perception and behaviour that vary tremendously across the types of dementia and corresponding levels of impairment, making the use of questionnaires and scales as data gathering tools unreliable or invalid. Qualitative research has mostly relied on structured interviews and observations, with inherent assumptions about a person’s capacity to verbally respond to questions and reflect on recent activities, both of which greatly diverge across the dementias. Underpinning this is the often-unspoken assumption by some researchers and clinicians that people with a dementia are not creative, nor can they continue to learn or participate meaningfully in new activities (Bellas et al., 2018).

More recently arts-related programmes in dementia care have been recommended for health and social care, charities and local communities to implement (e.g., All Party Parliamentary Group on Arts, Health and Wellbeing [APPG], 2017). Research relating to artistic creativity in the dementias has tended to focus on understanding the participatory aspects of specific art activities (e.g., Zeilig et al., 2014; Camic et al., 2016; Unadkat et al., 2017; Windle et al., 2017) within the context of healthcare or public health outcomes. However, in order to fully appreciate the complexity and potential of artistic creativity across different art forms, types of dementia, contexts most suitable to enhance and stimulate creativity, as well as approaches to measurement, we believe it is essential to conceptualise creativity in the dementias as a process that is not solely dependent on cognitive aptitude or skills, and to free it from the domain-general vs. domain-specific dichotomy that is “one of the most enduring controversies” in creativity research (Plucker and Beghetto, 2004, p. 153).

Going beyond this debate, considerable evidence from non-dementia research supports the idea that creativity has both specific *and* general components, yet a third component, the social environment of the individual (Amabile, 2013) is also fundamental to a conceptualisation of creativity in dementia. For people with a dementia, the social environment can help foster creativity. In particular, co-creativity is characterised by social interaction between two or more people in a supportive environment (including: home, public space, community centre, residential care, palliative care).

Rather than seeing creativity as necessitating an end product, creativity in the dementias emphasises process and experience (Killick and Craig, 2012), whereas co-creativity adds components such as mutual endeavour, relational interactions and notions of shared creativity. The emphasis on artistic creative *process* rather than on creative *outcomes*, is a necessary shift away from pre – post measurement of specific variables at given points in time. This shift allows new forms of measurement to be considered, such as obtaining continuous psychophysiological measures of specific moments in time; undertaking longitudinal ethnographic research looking at both the development of and changes in creativity; using eye tracking devices to better understand what is being seen in the moment; investigating the relationship between creative activity and wellbeing (e.g., Strohmaier and Camic, 2017). Emphasis on process over outcomes we argue, is also a more ethical way to research artistic creativity in individuals with a dementia because it places less emphasis and demand on production and end point measurement, whilst giving more attention to encouraging enjoyment, collaboration, exploratory trial and error and discovering what is possible, rather than establishing what is not.

## Ethics Statement

This projects reported in this article were carried out in accordance with the recommendations of the British Psychological Society research guidelines and approved by faculty ethics committees at University College London and Canterbury Christ Church University. All subjects gave written informed consent in accordance with the Declaration of Helsinki.

## Author Contributions

PC developed the idea for the article, which was further discussed and refined by SC, CM, EH, CH, SS, JW, GW, and HZ. All authors contributed sections including revisions. HZ, GW, SC, and PC critically revised further draughts. All authors read and approved the final manuscript.

## Conflict of Interest Statement

The authors declare that the research was conducted in the absence of any commercial or financial relationships that could be construed as a potential conflict of interest.

## Footnotes

1 http://www.createdoutofmind.org/
