# Supplementary material for: Conceptualising and Understanding Artistic Creativity in the Dementias: Interdisciplinary Approaches to Research and Practise
Source: Front Psychol. 2018 Oct 3;9:1842. doi: 10.3389/fpsyg.2018.01842 (PMC6178924; doi:10.3389/fpsyg.2018.01842)
Supplement: Supplementary file 1 [file Table_1.DOCX]

Table 1 *Perspectives on creativity from those with a dementia and informal and formal caregivers*

**Meaning of artistic creativity**

- When I first got diagnosed, taking photos was a way of trying to remember. I still love taking photos of beautiful things. I also go to writing group weekly which is another way to be creative for me, and not lose those skills, like handwriting.
- It is the golden thread that connects us all... running through everything.
- We use a mix of art forms, but creativity is in our thinking, imagination and being-ness with people.

**A personal understanding**

- I see creativity as something that is creative out of the mind, like painting, photography, writing, etc. Imagination. What I think is beautiful, someone else might see it differently. It's also a way to express ourselves, good and bad.
- Creativity is a life force that can be expressed, shared or connected with. It is all inclusive... and has emotional significance.
- It’s responding to the unexpected in a way that helps you somehow.

**The bidirectional impact of creativity on dementia**

- I don't think creativity impacts on dementia or vice versa. We all have images in our head, some good some bad. I see my dementia as being a black entity that’s dark, whereas me, myself I am light.
- As cognitive function changes, or words disappear, or confusion increases, we are left with creative and emotional ways to communicate with one another. The capacity to express ourselves is important in identity/changing identity.
- People with a diagnosis of dementia often express a range of ideas, thoughts, emotions and meaning through the arts...through to end of life.
- We note the impact their art has on families, Doctors, care staff and the general public, such as the realisation that people with dementia have ideas to express, and that it behoves us all to listen and observe.

**Creativity as a powerful process**

- When I wrote my poetry about dementia, it was cathartic. It wasn't always positive, it was a way to get all those feelings out of me, to get understanding that I lost some part of me somewhere.
- Does a creative process ever cause upset? Yes, it can. A creative process can trigger a range of emotions, some due to poignant memories, some through an expression of loss or frustration.
- Creativity is a life force that can be expressed, shared or connected with. It has emotional significance.
